# Editorial: Dietary influences on white and brown adipose tissue: mechanisms and health implications

**DOI:** 10.3389/fnut.2025.1731532

**Published:** 2025-11-20

**Authors:** Marcela Reymond Simoes, Bruna Bombassaro, Milena Monfort-Pires

**Affiliations:** 1 Obesity and Comorbidities Research Center, Institute of Biology, University of Campinas (UNICAMP), Campinas, São Paulo, Brazil; 2 Turku PET Centre, University of Turku, Turku, Finland; 3 Turku PET Centre, Turku University of Hospital, Turku, Finland

**Keywords:** adipose tissue, diet, nutrients, bioactive compounds, metabolic health

## Introduction

Obesity is a primary global concern, with 2.5 billion adults living with overweight and 890 million living with obesity worldwide (1). Although new pharmaceutical approaches have emerged in recent years to combat this epidemic, the diverse phenotypes, multifactorial and chronic nature of the condition, and the high prevalence of weight regain highlight the need for continuous research into alternative and adjuvant treatments, as well as a better understanding of the disease (2, 3). Excessive caloric intake and reduced energy expenditure due to physical inactivity are well-established contributors to fat accumulation (4, 5).

Over the past two decades, brown adipose tissue (BAT) has gained significant attention for its critical role in metabolism and overall health (6–8). When activated, BAT consumes circulating metabolites to generate heat (9, 10) and releases signaling molecules that exert systemic benefits (11, 12). A recent retrospective study reported that active BAT is associated with lower odds of cardiometabolic diseases (6). In contrast, white adipose tissue (WAT) primarily stores energy and secretes hormones that regulate metabolism and inflammation (13). Excessive WAT accumulation, especially in visceral depots, contributes to chronic inflammation and metabolic dysfunction (14). Both BAT and WAT therefore act as essential endocrine organs that maintain energy balance and body homeostasis.

As nutrition extends beyond caloric balance, increasing attention has been given to the role of nutrients in understanding and treating obesity. This Research Topic examines how nutrients and bioactive compounds influence adipose tissue biology. The collected manuscripts and review provide valuable insights into the dietary modulation of BAT activation and WAT metabolism.

## The impact of dietary factors on the function of adipose tissues

Diet is one of the most important modifiable risk factors for obesity and cardiometabolic diseases, as it directly influences adipose tissue function (15, 16). However, the specific effects of foods, nutrients, and bioactive compounds on adipose tissue remain underexplored (17, 18), particularly in humans. Nevertheless, growing evidence suggests that supplementation with micronutrients and bioactive compounds can modulate the function of WAT and BAT (17–19), influencing thermogenesis and inflammation.

The interest in knowing which food components have a greater impact on metabolic health also led to the development of diets for mouse models rich in sugar and/or saturated fat. Weiner et al. explored the effects of these diets on female mice, an important advance in the scientific field since only male mice were used until a few years ago. They showed that both diets had a negative impact on their health. The high-fat diet was more inflammatory for adipose tissue, impairing glucose homeostasis and insulin resistance. At the same time, high sucrose showed more prominent effects on the liver, including inflammation and lipid accumulation.

Understanding how food components enhance brown adipocytes' function could serve as a tool to manage metabolic diseases and obesity. This was explored in the review written by Bombassaro et al. In this review, the authors consolidate knowledge on food components related to mouse and human brown adipocyte differentiation and activation, describing how these nutrients act on this cell type, with emerging evidence on their impact on thermogenesis and health.

## The effects of nutrients and bioactive compounds on adipose tissue

Studies investigating the effects of nutrients and bioactive compounds have been conducted primarily in rodent models or cell cultures due to safety and dosage limitations (17, 18). Compounds such as capsaicin, curcumin, resveratrol, anthocyanins, and menthol have been shown to promote WAT browning/beiging and reduce inflammation (17, 18). Jiang et al. explored the correlation between red blood cell (RBC) folate, a type of vitamin B, and the amount of visceral fat. Using data from young and middle-aged adults from the NHANES database, the authors described how RBC folate levels exhibit a threshold effect, showing a negative correlation with visceral fat for values below 397.18 ng/mL and a positive correlation for values above. The rise of RBC folate as a marker for metabolic risk is highly relevant for clinical purposes, as it is a more straightforward and cost-effective biomarker than computed tomography (CT) or magnetic resonance imaging (MRI).

Vinnai et al. investigated the role of vitamin B1 (thiamine) supplementation in the *in vitro* differentiation of brown adipocytes from the human classic BAT depot and subcutaneous white adipose tissue. They showed that thiamine increased the expression of browning-related genes and respiratory capacity in both cell types, increasing their metabolism. The authors also discussed that thiamine deficiency in humans causes symptoms such as hypothermia and hypothalamic lesions, which are related to the functioning and regulation of brown adipose tissue. Thiamine deficiency is also seen in patients with type 1 and type 2 diabetes, making thiamine a micronutrient of great interest for supplementation to improve metabolic health.

Moreover, a study conducted by Guimarães et al. investigated the role of *Citrus aurantium*, popularly known as bitter orange, combined with synephrine, a β3 adrenergic receptor agonist, in metabolic health in a mouse model of childhood obesity. This intervention prevented weight gain and hyperleptinemia, reduced excessive fat accumulation in brown adipose tissue, and increased UCP1 expression in the mouse model. As mentioned above, the use of β3 adrenergic receptor agonists to activate BAT can be accompanied by increased heart rate and blood pressure; however, this was not observed on the combined intervention of synephrine and *Citrus aurantium*.

## Conclusion

This Research Topic provides essential information on the influence of diet on WAT and BAT. The gathered articles show the current knowledge on the impact of nutrients and food components in BAT and Beige activation, the adverse effect of diets rich in fat and sugar on metabolism, and the possibility of using micronutrients and food components, such as thiamine, folate, and *Citrus aurantium*, as supplements to improve adipose tissue metabolism and metabolic health. Therefore, the findings from this research can contribute to the study of new strategies to prevent and treat obesity and its comorbidities, improving the overall metabolic health of the current and future generations.
